# Affinity for risky behaviors following prenatal and early childhood exposure to tetrachloroethylene (PCE)-contaminated drinking water: a retrospective cohort study

**DOI:** 10.1186/1476-069X-10-102

**Published:** 2011-12-02

**Authors:** Ann Aschengrau, Janice M Weinberg, Patricia A Janulewicz, Megan E Romano, Lisa G Gallagher, Michael R Winter, Brett R Martin, Veronica M Vieira, Thomas F Webster, Roberta F White, David M Ozonoff

**Affiliations:** 1 Department of Epidemiology, Boston University School of Public Health, Talbot 3E, 715 Albany Street, Boston, MA 02118, USA; 2 Department of Biostatistics, Boston University School of Public Health, Crosstown, 715 Albany Street, Boston, MA 02118, USA; 3 Department of Epidemiology, University of Washington, Box 357236, Seattle WA, 98195, USA; 4 Department of Environmental Health, Boston University School of Public Health, Talbot 4W, 715 Albany Street, Boston, MA 02118, USA; 5 Data Coordinating Center, Boston University School of Public Health, Crosstown, 715 Albany Street, Boston MA 02118, USA; 6 Department of Neurology, Boston University School of Medicine, 72 East Concord Street, Boston Ma 02118 USA

## Abstract

**Background:**

Many studies of adults with acute and chronic solvent exposure have shown adverse effects on cognition, behavior and mood. No prior study has investigated the long-term impact of prenatal and early childhood exposure to the solvent tetrachloroethylene (PCE) on the affinity for risky behaviors, defined as smoking, drinking or drug use as a teen or adult.

**Objectives:**

This retrospective cohort study examined whether early life exposure to PCE-contaminated drinking water influenced the occurrence of cigarette smoking, alcohol consumption, and drug use among adults from Cape Cod, Massachusetts.

**Methods:**

Eight hundred and thirty-one subjects with prenatal and early childhood PCE exposure and 547 unexposed subjects were studied. Participants completed questionnaires to gather information on risky behaviors as a teenager and young adult, demographic characteristics, other sources of solvent exposure, and residences from birth through 1990. PCE exposure was estimated using the U.S. EPA's water distribution system modeling software (EPANET) that was modified to incorporate a leaching and transport model to estimate PCE exposures from pipe linings.

**Results:**

Individuals who were highly exposed to PCE-contaminated drinking water during gestation and early childhood experienced 50-60% increases in the risk of using two or more major illicit drugs as a teenager or as an adult (Relative Risk (RR) for teen use = 1.6, 95% CI: 1.2-2.2; and RR for adult use = 1.5, 95% CI: 1.2-1.9). Specific drugs for which increased risks were observed included crack/cocaine, psychedelics/hallucinogens, club/designer drugs, Ritalin without a prescription, and heroin (RRs:1.4-2.1). Thirty to 60% increases in the risk of certain smoking and drinking behaviors were also seen among highly exposed subjects.

**Conclusions:**

The results of this study suggest that risky behaviors, particularly drug use, are more frequent among adults with high PCE exposure levels during gestation and early childhood. These findings should be confirmed in follow-up investigations of other exposed populations.

## Background

In 1980 government officials in New England learned that tetrachloroethylene (PCE) was leaching into public drinking water supplies from the vinyl lining (VL) of asbestos cement (AC) water distribution pipes. The liner, which was used since the late 1960s to address alkalinity problems, had been sprayed onto the inner pipe surface in a slurry of vinyl toluene resin and PCE. Because the pipes were allowed to dry for 48 hours before shipping [1] it was assumed that most of the PCE had evaporated by the time the pipes were installed. However, more than a decade passed before officials discovered that sizeable quantities of PCE had remained in the lining and were leaching into the drinking water supplies.

Surveys done after the discovery of contamination estimated that approximately 660 miles of VL/AC pipes had been installed in nearly 100 cities and towns in Massachusetts [2]. Because VL/AC pipes were installed in response to replacement and expansion needs in a town's water system, the contamination pattern was irregular and neighboring residents often had vastly different exposure levels. PCE levels in water samples taken in 1980 ranged from 1.5 to 80 ug/L in medium and high flow locations and 1,600 to 7,750 ug/L in low-flow locations such as dead end streets [1]. Replacing the VL/AC pipes was prohibitively expensive, and so officials initiated a program of flushing and bleeding to reduce levels below 40 μg/l, the action level in 1980 based on the U.S. EPA Suggested No Adverse Response Level (SNARL) [1]. Currently, the maximum contaminant level for PCE is set at 5 μg/L. Exposure to PCE from drinking water occurs by direct ingestion, dermal exposure during bathing and, because it volatilizes easily, by inhalation during showering, bathing and other household uses.

PCE is a well-recognized animal and human neurotoxicant [3]. Many epidemiologic studies of adults with occupational exposure to PCE and other solvents report impairments in cognition, memory, attention, and executive function [4-9]. Mood changes, including increases in anxiety and depression, have also been reported [4,6,7,10,11].

The published literature examining neurotoxic effects among children with prenatal and childhood exposure to organic solvents including PCE, on the other hand, is comparatively small and results are not consistent [12]. Four studies found no deficits in cognitive and behavioral function or no increases in disorders of attention and learning [13-16], while two found lower tests scores and more behavioral problems among children with prenatal or early childhood exposure to organic solvents [17,18]. No prior studies have examined possible long-lasting neurological consequences of early life exposure. Thus, we undertook a population-based retrospective cohort study to examine the long-term neurotoxic effects of prenatal and early childhood exposure to PCE contaminated drinking water. The current report focuses on the affinity for three risky behaviors: cigarette smoking, alcoholic beverage consumption, and drug use as a teenager and adult. The suspicion that prenatal and early childhood exposure to PCE might increase the occurrence of these behaviors stems from reports that prenatal exposure to another organic solvent --alcohol-- increases the risk of these behaviors during adolescence and early adulthood [19,20].

## Methods

### Selection of Study Population

Subjects were eligible for the study if they were born during 1969-1983 to married women living in Cape Cod, Massachusetts towns known to have some VL/AC water pipes installed in some parts of their water distribution system. Such a study subject is called an "index subject." Eight towns in the study area were affected --Barnstable, Bourne, Falmouth, Mashpee, Sandwich, Brewster, Chatham, and Provincetown--each having anywhere from one to 50 miles of VL/AC pipes [2]. Eligible subjects were identified by reviewing more than 13,000 birth certificates and cross-matching the maternal address on the certificate with information collected from water companies on the location, installation year, and diameter of all VL/AC pipes in the water distribution system.

Initially, two groups of index subjects were selected: (1) those born to women who were exposed to PCE-contaminated drinking water at birth, and (2) those born to women who were unexposed at their birth. Index subjects were initially designated as "exposed" because their birth residence was either directly adjacent to a VL/AC pipe or because the residence was adjacent to a pipe connected to a VL/AC pipe and the only possible water flow to the residence was through the VL/AC pipe. Thus, the initial exposure status was based on a visual inspection of maps depicting the pipe distribution network in the immediate vicinity of the birth address. The initial "exposed" group included 1,910 subjects. A comparison group initially designated as "unexposed" was randomly selected from the remaining resident births during this period. "Unexposed" subjects were frequency matched to "exposed" subjects on month and year of birth. The initial "unexposed" group included 1,928 subjects.

In addition, 1,202 older siblings of the "index" subjects were identified for the present study. Only siblings born in Massachusetts during 1969-1983 were eligible for selection. All older siblings were initially considered "unexposed" at birth because they were born before the family moved to an affected Cape Cod residence. However, the initial exposure status of all subjects was considered tentative until more extensive exposure assessments, as described below, were completed.

Birth certificates of all subjects were reviewed to obtain information on the study family, including the full names of the subject and parents; the subject's date of birth, birth weight and gestational duration; and the parents' ages and educational levels when the subject was born. All mothers were married at the time of the index subject's birth. The study was approved by the Institutional Review Boards (IRB) of the Massachusetts Department of Public Health and Boston University Medical Center and by the 24A/B/11B Review Committee at the Massachusetts Department of Public Health.

### Follow-Up and Enrollment of Study Subjects

Follow-up and enrollment of subjects occurred during 2006-2008. Subjects were traced to obtain their current address and telephone number using resources such as Massachusetts Resident's Lists; death, credit bureau, and alumni records; telephone books, directory assistance, and the Internet White Pages. Letters were sent to all successfully traced subjects describing the general purpose of the study and requesting that they complete a self-administered questionnaire. Three follow-up letters were sent to non-respondents, and subjects who did not respond to these letters were phoned if their telephone number was available. As described in Table 1, 6.6% of the selected population could not be located (n = 332), 45.5% were located but never responded to any of our contact attempts (n = 2,294), 3.7% refused to participate (n = 187), and 2.2% were deceased (n = 111). In addition, the Massachusetts Department of Public Health IRB did not allow us to contact another 8.5% of subjects whose parent had refused to participate in our prior cohort study of reproductive and developmental outcomes (n = 427, Aschengrau et al, 2008). These percentages were similar for both "exposed" and "unexposed" index subjects and their older siblings.

**Table 1:** Selection, enrollment, and initial and final exposure status of study subjects

	Initial Exposure Status			
	Index Subject		Older Sibling	
	Exposed	Unexposed	Unexposed	Total
Selected	1910	1928	1202	5040
Excluded during enrollment				
Deceased	35	40	36	111
Parent refused participation	199	148	80	427
Never located	113	149	70	332
No response	871	887	536	2294
Refused	73	78	36	187
Returned questionnaire	619	626	444	1689
Percent of selected	32.4%	32.5%	36.9%	33.5%
Percent of located	39.6%	39.3%	43.7%	40.5%
Excluded during exposure assessment				
Inadequate residential history	15	37	29	81
Off-Cape address in potential VL/AC town	19	27	50	96
Available for analysis	585	562	365	1512
Percent of selected	30.6%	29.1%	30.4%	30.0%
Final exposure status				
Both prenatal and early childhood exposure	561	160	110	831
Only early childhood exposure	7	42	85	134
Unexposed	17	360	170	547
				

We conducted analyses comparing available characteristics among participants and non-participants. Most obtainable characteristics were similar, including initial PCE exposure status (36.7% of participants vs. 38.8% of non-participants were exposed), race (98.5% of participants vs. 97.3% of non-participants were White), age (mean age as of June 30, 2007 was 29.3 years for participants and 28.9 years for non-participants), birth order (47.7% of participants vs. 45.1% of non-participants were first born), mean duration of gestation (40.1 weeks for participants vs. 39.9 weeks for non-participants) mean birth weight (3,428 grams for participants vs. 3,401 for non-participants), While participants were more likely to be female (60.7% of participants vs. 43.5% of non-participants), and have mothers with some college education (61.0% for participants vs. 49.3% for non-participants), these differences were present for both exposed and unexposed non-participants.

A self-administered questionnaire was sent to all successfully traced subjects to gather information on cigarette smoking, alcoholic beverage consumption, and drug use. In particular, information was obtained on: (1) ever becoming a regular cigarette smoker, age at initiation of smoking on a regular basis, and smoking frequency and amount during the past 30 days; (2) ever consuming an alcoholic drink (defined as a 12 ounce bottle, can or glass of beer; 12 ounces of wine cooler, hard lemonade, or hard cider, a 4 ounce glass of wine, or a shot or liquor straight or in a mixed drink); age at initiation of drinking, typical drinking frequency and amount as a teenager (ages 13 to 18 years), and drinking frequency and amount during the past 30 days; and (3) ever use of marijuana, inhalants, heroin, cocaine or crack, psychedelics or hallucinogens, Ritalin without a prescription, and club drugs as a teenager (ages 13 to 18 years) and adult (ages 19+ years).

The questionnaire also gathered information on the subject's demographic characteristics including race, ethnicity, current marital status, educational level and occupation; personal history of learning disabilities and mental disorders; family history of learning disabilities and mental disorders; and occupational and non-occupational exposure to solvents. Information was also collected on the family's residences from the subject's birth through 1990, including the exact street address and calendar years of residence for all Cape Cod addresses. Lastly, the survey collected information on the subject's knowledge of the PCE exposure episode and self-assessments of their PCE exposure.

Additional questionnaire data were also available for 81% of subjects whose mothers participated in our prior cohort study on the impact of PCE exposure on reproduction and development. These data included maternal characteristics, such as changes in marital status; cigarette smoking, alcoholic beverage consumption, marijuana use, medical and obstetrical complications, and prenatal care during the subject's gestation; breast feeding practices; and exposure to solvents. Information on the mother's water use patterns during the subject's gestation and childhood was available for only 58% of subjects and the prevalence of key variables was relatively low (e.g., only 20% of mothers stated that they regularly drank bottled water during the subject's gestation) and so these data were not incorporated into our present analyses.

### Geocoding of Residential Addresses

All residential addresses on Cape Cod reported by subjects were geocoded to a latitude and longitude using ArcGIS 8.1. Whenever possible each address was geocoded to a parcel of land using information from the questionnaire, county deeds, assessors' maps, town voter registration lists, and Internet resources. Addresses that could not be geocoded to a specific parcel were geocoded to the closest parcel address by street number. If a street number was unavailable and the street was less than a mile long, the address was geocoded to the middle of the street. If the street was a mile or longer, the address was geocoded to the intersection of the address with the cross-street given in the survey. Approximately 95% of reported addresses were successfully geocoded. The remainder could not be geocoded because insufficient information was provided by the subject. Geocoding was done without knowledge of the exposure or outcome status.

### PCE Exposure Assessment

At the start of the study, we assigned an initial tentative exposure status to each subject, as described above, after visually inspecting maps of the pipe distribution network in the immediate vicinity of the birth residence. To determine the final exposure designation, we used a leaching and transport model to estimate the mass of PCE delivered to each residence from the prenatal period through the age of five years. The model, which was developed for our prior epidemiological studies by Webler and Brown [21,22], estimates the quantity of PCE entering the drinking water using the initial amount of PCE in the liner, the age of the pipe, and the leaching rate of PCE from the liner into the water. The pipe's initial stock of PCE was based on the pipe's diameter and length. Laboratory experiments by Demond suggested that the leaching process followed a simple exponential relationship with rate constant of 2.25 years [1]. Thus, PCE drinking water levels shortly after a VL/AC pipe was installed were much higher than levels a few years later.

The transport algorithm requires an estimate of water flow rate and direction, which are functions of the configuration of the pipes and number of water users. The present study incorporated the Webler and Brown algorithm into the publically available source code of the EPANET water distribution modeling software to characterize water flow throughout a town's entire public distribution system. EPANET, which was developed by the U.S. EPA to help water utilities design water monitoring programs [23] has been applied in several other epidemiological studies assessing the health effects of drinking water contaminants [24-27], and because its source code is public ("open source") we were able to add the leaching and transport component.

We used maps of subject residences and the distribution systems to create a schematic depicting the water sources, pipe characteristics, and nodes, representing points of water consumption along the pipe. Information on the locations, installation dates and diameters of all VL/AC pipes in the public water supply were obtained from local water departments and the Massachusetts Department of Environmental Protection (DEP).

We used the schematic to assign each residence to the closest node on the distribution system. We assumed that all users drew the same amount of water because the study area was mainly comprised of residences. Based on information in available records, we also assumed that water sources did not change over the study period. The distribution systems that were in place in the 1960s and 1970s remained generally unchanged until population growth required some expansion in the 1980s.

The model incorporated these data to simulate the instantaneous flow of water through each town's network and to estimate the mass of PCE delivered to each node and all subject residences associated with the node for each year of the study period. We were able to calculate only annual PCE exposures because only move-in and pipe installation years were available. We estimated PCE exposure during the prenatal period by multiplying the annual mass of PCE that entered the subject's residence during their birth year by 9/12. We estimated cumulative exposure during early childhood by summing the estimated mass of PCE that entered their residences from the month and year following birth through the month and year of the fifth birthday. Simple percentages were used to account for partial years.

We estimated PCE exposure levels only for subjects with complete geocoded residential histories from birth through age five. As seen in Table 1, a total of 81 subjects were excluded because they had inadequate residential histories. Another 96 subjects were excluded because one or more of their residences was in an off-Cape town with some VL/AC pipe and our PCE exposure assessments were limited to Cape Cod. Subjects who reported living in a Cape Cod town without any VL/AC pipes (n = 7) were assumed to have no PCE exposure at that address. This was considered a reasonable assumption because available records from this geographic area and time period indicated little or no PCE contamination of these water sources.

### Statistical Analysis

We compared the occurrence of each risk-taking behavior among subjects with prenatal and early childhood exposure combined to unexposed subjects. First, we examined the impact of any PCE and then divided the exposure into tertiles to examine a dose-response relationship. Nearly all subjects with prenatal exposure also had childhood exposure and so we were unable to examine the impact of prenatal exposure only. In addition, we did not examine the impact of exposure only during childhood because there were too few subjects in this category (n = 134) to provide stable effect estimates. For the cigarette smoking outcomes, we examined ever becoming a regular smoker, initiating smoking on a regular basis at a young age (defined as 13 years or under), and becoming a heavy smoker as an adult (defined as 20+ cigarettes per day in the past 30 days) in comparison to never becoming a regular smoker. For drinking alcoholic beverages, we examined initiating drinking at a young age (13 or younger vs. 14 and older), high frequency drinking as a teen (more than eight days a month vs. never drinking as a teen), and heavy drinking as a teen (five or more drinks per day for males and four or more drinks per day for females vs. never drinking as a teen). In addition, we examined high frequency drinking as an adult (more than eight days a month in past 30 days) and heavy drinking as an adult (five or more drinks per day for males and four or more drinks per day for females in past 30 days), all in comparison to not drinking in the past 30 days. For illicit drug use, we examined separately ever use of marijuana, inhalants, heroin, crack/cocaine, psychedelics/hallucinogens, Ritalin without a prescription, and club drugs/designer drugs as a teen and adult in comparison to never using any drugs at any time. Inhalants included glue, paint, gasoline, and cleaning solutions; psychedelics/hallucinogens included "LSD," "acid," "PCP," and mescaline; and club/designer drugs included "Ecstasy," "XTC," "Special K," "GHB," and "Liquid X." In addition, we examined the following groupings of illicit drug use as a teenager and adult: any drugs, any major drugs (which included all drugs, except marijuana), two or more drugs, and two or more major drugs in comparison to never using any drugs at any time. Lastly, we assessed the association between PCE exposure and multiple risky behaviors by examining the combinations of initiating drinking at a young age, high frequency of drinking, and major drug use as a teenager and heavy smoking, heavy drinking, and major drug use as an adult.

The risk ratio (RR) was used to estimate the strength of the association between PCE exposure and the occurrence of each behavior. Ninety-five percent confidence intervals were used to assess the precision of the risk ratios. Crude analyses were conducted on the entire study population and repeated on a subset of subjects whose mothers reported that they did not smoke cigarettes or marijuana, or drink alcoholic beverages while they were pregnant with the study subject. Next, generalized estimating equation (GEE) analyses were performed to account for non-independent outcomes arising from several children from the same family [28,29]. Thirty-nine percent of the children in the study were siblings. The logit link was used while assuming equal correlation between birth outcomes from the same mother.

Lastly, adjusted GEE analyses were conducted to control for confounding variables. Covariates considered for these analyses were demographic characteristics, factors associated with risk-taking behavior (e.g., maternal alcoholic beverage consumption when she was pregnant with the subject), and non-drinking water sources of solvent exposure. These variables included the subject's gender, race, age when the study questionnaire was completed, age at onset of puberty, breast feeding status, and history of learning disabilities and mental illness; the mother's age and educational level when the subject was born; paternal age, educational level and occupation when the subject was born; the mother's prenatal care, multivitamin use, alcoholic beverage consumption, cigarette smoking, marijuana use, medical conditions, and obstetrical complications when she was pregnant with the subject; number of prior live-born siblings; death of a sibling; and maternal history of solvent exposure. In order to determine which variables to control in the adjusted analyses, we first assessed how strongly each variable was associated with PCE exposure. Categorical variables were retained for further consideration if there was more than a 5% difference in the frequency of a characteristic between compared groups. Variables that met these criteria were maternal educational level and paternal occupation at the subject's birth, number of prior live-born siblings, and the subject's breast feeding status. For example, there was a 5.2% difference between exposed and unexposed subjects in the proportion of mothers with some college education. Each of these variables was entered into the crude GEE models one at a time. However, because none of these variables changed the crude GEE risk ratio by at least 10%, no multivariate analyses were conducted.

## Results

A total of 1,512 subjects was available for the analysis. According to the initial exposure designation, there were 585 exposed and 562 unexposed index subjects and 365 unexposed older siblings (Table 1). Following the more refined EPANET exposure assessment, 397 unexposed index subjects and older siblings changed their status to exposed. These subjects had birth addresses that were further downstream from a VL/AC pipe than was originally considered exposed in the initial visual assessment. The modeled assessment of the entire distribution system indicated that these downstream locations had PCE contamination. In addition, 17 exposed index subjects changed their status to unexposed. These were either subjects whose modeled assessment indicated a different water flow direction than was originally assumed or subjects whose questionnaire data indicated that they used private wells as their water supply. The final analytic population was comprised of 831 subjects with both prenatal and early childhood exposure and 547 subjects with no exposure during either period. The latter unexposed group included 377 (= 17 + 360) index subjects and 170 older siblings.

As shown in Table S1 (see Additional file 1), the characteristics of the exposed and unexposed subjects were quite similar. Subjects were predominantly female, white, college-educated, in their late 20s, married or cohabitating, and employed when they completed the study questionnaires. Few subjects had possible occupational exposure to solvents but many had potential exposure from hobbies and other sources. Comparable proportions of exposed and unexposed index subjects also reported a personal history of learning disabilities, repeating a grade, and mental disorders (mainly depression), and a family history of mental disorders (also mainly depression). Parental characteristics were also similar, including their age, educational level and occupation when the subject was born. In addition, comparable proportions of mothers reported smoking cigarettes and marijuana, drinking alcoholic beverages, having prenatal care, and a history of medical or obstetrical complications when they were pregnant with the study subjects. The occurrence of parental divorce and the death of a sibling were also comparable.

The study population experienced a wide distribution of PCE exposure levels that encompassed several orders of magnitude (Table S2, Additional file 2). Cumulative prenatal exposure levels were lower than early childhood levels because of their different durations (i.e., nine months vs. five years). Mean (SD) exposures were 32.6 (88.6) and 109.0 (283.3), respectively, for prenatal and early childhood exposure.

As described, the exposure measures were based on the mass of PCE delivered to a subject's residence through the water distribution system over the course of a year. This annual mass of PCE was diluted in approximately 90,000 gallons of water, the average annual usage for a family of four [30]. When we converted our PCE exposure measures to annual point concentrations, we estimated that PCE concentrations in water entering the study homes ranged from less than 1 ug/L to 5,197 ug/L. These levels are consistent with public water testing results during the study period [1].

Our comparison of each subject's self-assessed exposure status to that derived from the modeled assessment revealed that only 7% of subjects considered exposed by the modeled assessment thought that their drinking water was contaminated, whereas 29% of these exposed subjects thought that their water was not contaminated and 64% were unsure. Similarly, we found that 31% of subjects considered unexposed by the modeled assessment thought that their drinking water was not contaminated while 5% thought that their drinking water was contaminated and 63% were unsure.

Associations between PCE exposure and each risk-taking behavior are described below and are summarized in Table 2 within the text. Analyses of the association between any PCE exposure and cigarette smoking did not reveal any meaningful increases in the risk of becoming a regular smoker, initiating smoking at a younger age, or smoking heavily in the past 30 days among subjects with any exposure during gestation and early childhood (Table 2; Table S3, Additional file 3). However, when exposure levels were examined, small increases in the risk of becoming a regular smoker (RR: 1.2, 95% CI: 1.0-1.5), initiating smoking at a younger age (RR: 1.4, 95% CI: 0.8-2.5) and heavy smoking (RR: 1.3, 95% CI: 0.9-2.0) were observed among subjects in the highest exposure tertile.

**Table 2:** Prenatal and Early Childhood Exposure to Tetrachloroethylene and Selected^1 ^Risky Behaviors

Outcome	Exposure Category/ Percentile	% Yes (n/N)	Crude RR (95% CI)	Simple GEE RR (95% CI)
Ever smoked regularly vs. Never smoked regularly^2^	Any	36.9 (303/821)	1.0 (0.9-1.2)	1.0 (0.9-1.2)
	> = 67^th^	45.0 (122/271)	1.3 (1.1-1.5)	1.2 (1.0-1.5)
	33^rd^- < 67^th^	29.1 (81/278)	0.8 (0.7-1.0)	0.8 (0.7-1.0)
	> 0- < 33^rd^	36.8 (100/272)	1.0 (0.9-1.3)	1.0 (0.9-1.3)
	None	35.2 (191/543)	Reference	Reference
				
Smoked 20+ cigarettes a day vs. Never smoked regularly^3^	Any	12.6 (75/593)	1.1 (0.8-1.5)	1.1 (0.8-1.5)
	> = 67^th^	15.8 (28/177)	1.3 (0.9-2.0)	1.3 (0.9-2.0)
	33^rd^- < 67^th^	12.1 (27/224)	1.0 (0.6-1.6)	1.0 (0.7-1.6)
	> 0-< 33^rd^	10.4 (20/192)	0.9 (0.5-1.4)	0.9 (0.5-1.4)
	None	12.0 (48/400)	Reference	Reference
				
First drank at < = 13 years vs. 14+ years^4^	Any	19.6 (159/811)	1.1 (0.8-1.3)	1.1 (0.8-1.3)
	> 67^th^	21.9 (59/269)	1.2 (0.9-1.6)	1.2 (0.9-1.6)
	33^rd^- < 67^th^	18.4 (51/277)	1.0 (0.7-1.3)	1.0 (0.7-1.4)
	> 0-< 33^rd^	18.5 (49/265)	1.0 (0.7-1.4)	1.0 (0.7-1.4)
	None	18.6 (98/528)	Reference	Reference
				
Drank > 8 days/mo as teen vs. Never drank as a teen^5^	Any	26.2 (68/260)	1.1 (0.8-1.5)	1.1 (0.8-1.5)
	> 67^th^	38.8 (33/85)	1.6 (1.1-2.4)	1.6 (1.1-2.3)
	33^rd ^- < 67^th^	19.8 (18/91)	0.8 (0.4-1.5)	0.8 (0.5-1.3)
	> 0-< 33^rd^	20.2 (17/84)	0.9 (0.5-1.4)	0.8 (0.5-1.4)
	None	23.7 (42/177)	Reference	Reference
				
Drank > = 5/4 drinks/drinking day in past 30 days	Any	47.2 (133/282)	1.1 (0.9-1.3)	1.1 (0.9-1.3)
vs. Did not drink in past 30 days^6^	> 67^th^	57.6 (53/92)	1.3 (1.0-1.7)	1.3 (1.0-1.7)
	33^rd ^- < 67^th^	41.4 (36/87)	1.0 ((0.7-1.3)	0.9 (0.7-1.3)
	> 0-< 33^rd^	42.7 (44/103)	1.0 (0.7-1.3)	1.0 (0.7-1.3)
	None	43.5 (83/191)	Reference	Reference
				
2+ Major drugs as a teen vs. Never used any drugs^7,8^	Any	35.7 (124/347)	1.4 (1.0-1.8)	1.4 (1.0-1.8)
	> 67^th^	43.3 (45/104)	1.6 (1.2-2.2)	1.6 (1.2-2.2)
	33^rd ^- < 67^th^	30.8 (40/130)	1.2 (0.8-1.6)	1.2 (0.8-1.6)
	> 0-< 33^rd^	34.5 (39/113)	1.3 (0.9-1.8)	1.3 (0.9-1.8)
	None	26.5 (59/223)	Reference	Reference
				
Crack/cocaine as a teen vs. Never used any drugs ^7,9^	Any	29.2 (92/315)	1.6 (1.1-2.2)	1.6 (1.1-2.2)
	> 67^th^	38.5 (37/96)	2.1 (1.4-3.1)	2.1 (1.4-3.0)
	33^rd ^- < 67^th^	22.4 (26/116)	1.2 (0.8-1.9)	1.2 (0.8-1.9)
	> 0-< 33^rd^	28.2 (29/103)	1.5 (1.0-2.3)	1.5 (0.9-2.3)
	None	18.4 (37/201)	Reference	Reference
				
Psychedelics/Hallucinogens as a teen vs. Never used any drugs^7,9^	Any	41.8 (160/383)	1.2 (1.0-.1.5)	1.2 (1.0-1.5)
	> 67^th^	47.3 (53/112)	1.4 (1.1-1.8)	1.4 (1.1-1.8)
	33^rd ^- < 67^th^	36.6 (52/142)	1.1 (0.8-1.4)	1.1 (0.8-1.4)
	0-< 33^rd^	42.6 (55/129)	1.2 (1.0-1.6)	1.3 (1.0-1.6)
	None	34.1 (85/249)	Reference	Reference
				
Club/Designer Drugs as a teen vs. Never used any drugs^7,9^	Any	27.8 (86/309)	1.4 (1.0-2.0)	1.5 (1.0-2.1)
	> 67^th^	39.8 (39/98)	2.1 (1.4-3.0)	2.1 (1.5-3.1)
	33^rd ^- < 67^th^	16.7 (18/108)	0.9 (0.5-1.4)	0.9 (0.5-1.5)
	> 0-< 33^rd^	28.2 (29/103)	1.5 (1.0-2.2)	1.5 (0.9-2.3)
	None	19.2 (39/203)	Reference	Reference
				
Ritalin without a prescription as teen vs. Never used any drugs^7,9^	Any	23.6 (69/292)	1.5 (1.0-2.3)	1.5 (1.0-2.2)
	> 67^th^	33.7 (30/89)	2.2 (1.4-3.4)	2.1 (1.4-3.3)
	33^rd ^- < 67^th^	16.7 (18/108)	1.1 (0.6-1.8)	1.1 (0.6-1.8)
	> 0-< 33^rd^	22.1 (21/95)	1.4 (0.9-2.4)	1.3 (0.8-2.2)
	None	15.5 (30/194)	Reference	Reference
				
2+ Major drugs as an adult vs. Never used any drugs^7,10^	Any	44.5 (179/402)	1.3 (1.0-1.6)	1.3 (1.0-1.6)
	> 67^th^	53.9 (69/128)	1.6 (1.2-2.0)	1.5 (1.2-1.9)
	33^rd ^- < 67^th^	37.1 (53/143)	1.1 (0.8-1.4)	1.1 (0.8-1.4)
	> 0-< 33^rd^	43.5 (57/131)	1.3 (1.0-1.6)	1.2 (1.0-1.6)
	None	34.7 (87/251)	Reference	Reference
				
Crack/cocaine as an adult vs. Never used any drugs ^7,11^	Any	43.5 (172/395)	1.2 (1.0-1.5)	1.2 (1.0-1.5)
	> 67^th^	53.2 (67/126)	1.5 (1.2-1.9)	1.4 (1.1-1.8)
	33^rd ^- < 67^th^	34.3 (47/137)	0.9 (0.7-1.3)	0.9 (0.7-1.2)
	> 0-< 33^rd^	43.9 (58/132)	1.2 (0.9-1.6)	1.2 (0.9-1.6)
	None	36.2 (93/257)	Reference	Reference
				
Psychedelics/Hallucinogens as an adult vs. Never used any drugs^7,11^	Any	42.1 (162/385)	1.2 (1.0-1.5)	1.2 (1.0-1.5)
	> 67^th^	48.7 (56/115)	1.4 (1.1-1.8)	1.4 (1.1-1.8)
	33^rd ^- < 67^th^	33.8 (46/136)	1.0 (0.7-1.3)	1.0 (0.7-1.3)
	0-< 33^rd^	44.8 (60/134)	1.3 (1.0-1.7)	1.3 (1.0-1.7)
	None	34.7 (87/251)	Reference	Reference
				
Club/Designer Drugs as an adult vs. Never used any drugs^7,11^	Any	45.1 (183/406)	1.3 (1.1-1.6)	1.3 (1.1-1.6)
	> 67^th^	52.0 (64/123)	1.5 (1.2-1.9)	1.5 (1.2-1.9)
	33^rd ^- < 67^th^	38.8 (57/147)	1.1 (0.9-1.5)	1.1 (0.9-1.5)
	> 0-< 33^rd^	45.6 (62/136)	1.3 (1.0-1.7)	1.3 (1.0-1.7)
	None	34.4 (86/250)	Reference	Reference
				
Ritalin without a prescription as an adult vs. Never used any drugs^7,11^	Any	28.5 (89/312)	1.4 (1.0-2.0)	1.4 (1.0-2.0)
	> 67^th^	38.5 (37/96)	1.9 (1.3-2.8)	1.9 (1.3-2.8)
	33^rd ^- < 67^th^	19.6 (22/112)	1.0 (0.6-1.6)	1.0 (0.6-1.5)
	> 0-< 33^rd^	28.8 (30/104)	1.4 (1.0-2.2)	1.4 (0.9-2.1)
	None	20.0 (41/205)	Reference	Reference
				
Heroin as an adult vs. Never used any drugs^7,11^	Any	9.0 (22/245)	1.3 (0.7-2.6)	1.3 (0.7-2.6)
	> 67^th^	11.9 (8/67)	1.8 (0.7-4.1)	1.7 (0.7-4.1)
	33^rd ^- < 67^th^	5.3 (5/95)	0.8 (0.3-2.1)	0.8 (0.3-2.1)
	> 0-< 33^rd^	10.8 (9/83)	1.6 (0.7-3.6)	1.6 (0.7-3.6)
	None	6.8 (12/176)	Reference	Reference

1 Table includes key findings with more than five subjects in the referent group

2 Comparison excludes subjects who smoked 100+ cigarettes but never became regular smokers

3 Comparison excludes subjects who smoked < 20 cigarettes a day

4 Comparison excludes subjects who never drank as a teen

5 Comparison excludes subjects who drank < = 8 days/month as a teen

6 Comparison excludes subjects who drank < 5/4 drinks/drinking day in past 30 days

7 Referent group is comprised of subjects who never used drugs as a teen or an adult

8 Comparison excludes subjects who used only marijuana or one major drug as a teen

9 Comparison excludes subjects who used any other type of drug as a teen

10 Comparison excludes subjects who used only marijuana or one major drug as an adult

11 Comparison excludes subjects who used any other type of drug as an adult

PCE exposure had a similar relationship with drinking alcoholic beverages. There were no meaningful increases in the risk of initiating drinking at a young age, drinking frequently and heavily as a teen, and drinking frequently and heavily in the past 30 days among subjects with any exposure during gestation and early childhood (Table 2; Table S4, Additional file 4). However, small to moderate increases in the risk of drinking frequently as a teen (RR: 1.6, 95% CI: 1.1-2.3) and drinking heavily in the past 30 days (RR: 1.3, 95% CI: 1.0-1.7) were observed among subjects in the highest exposure tertile.

Stronger associations were observed between PCE exposure and drug use. Subjects with exposure during both gestation and early childhood had 1.5- to 1.6-fold increases in the risk of using crack/cocaine, club/designer drugs, Ritalin without a prescription as a teenager (Table 2; Table S5, Additional file 5), and 1.3- to 1.4-fold increases in the risk of using club/designer drugs, Ritalin without a prescription, and heroin as an adult (Table 2; Table S6, Additional file 6). Furthermore, subjects in the highest tertile of prenatal and postnatal exposure had greater increases in risk. In particular, there were 1.4- to 2.1-fold increases in the risk of using crack/cocaine, psychedelics/hallucinogens, club/designer drugs, Ritalin without a prescription, and heroin as a teenager (Table 2; Table S5, Additional file 5), and 1.4- to 1.9-fold increases in the risk of using crack/cocaine, psychedelics/hallucingens, club/designer drugs, Ritalin without a prescription, and heroin as an adult (Table 2; Table S6, Additional file 6).

Because use of these drugs often co-occurred in the same individual, we also observed increases in the risk of multiple drug use among exposed subjects (Table 2; Tables S5 and S6, Additional files 5 and 6). In particular, we found a 40% increase in the risk of using two or more major drugs as a teenager (95% CI: 1.0-1.8) among subjects with any prenatal and early postnatal exposure that rose to 60% among subjects in the highest exposure tertile (95% CI: 1.2-2.2). We also found a 30% increase in the risk of using multiple major drugs as an adult (95% CI: 1.0-1.6) among subjects with any prenatal and early postnatal exposure that increased to 50% among subjects in the highest exposure tertile (95% CI: 1.2-1.9).

As expected, there was a high degree of overlap between the teenager and adult drug users. For example, nearly 78% of the subjects who reported using major drugs as teens went on to use these drugs as adults. In contrast, 18% of subjects who reported that they did not use major drugs as teens became major drug users as adults. Contrary to expectations, the joint occurrence of smoking, drinking and drug use in the same individual was relatively uncommon. For example, only 7.4% of the study population (n = 99) reported engaging in two or three of the following activities as a teen: initiating smoking before 14 years, drinking more than 8 days a month, and using major drugs, and only 13.8% (n = 182) reported engaging in two or three of the following activities as an adult: heavy smoking, heavy drinking, and using major drugs. Only 1.5% of the study population (n = 20) reported engaging in all three activities as a teen and 1.6% (n = 21) reported engaging in all three activities as an adult. Only three subjects reported a history of all three activities as both a teen and an adult.

However, when we assessed the relationship between prenatal and childhood PCE exposure and the occurrence of multiple risky behaviors (Table S7, Additional file 7), we found a 2.7-fold increase in the risk (95% CI: 0.9-8.0) of engaging in the following behaviors as a teen: initiating smoking at < 14 years, drinking alcoholic beverages > 8 days/month, and using major drugs. This rose to a 5.4-fold increased risk among subjects in the highest exposure tertile (95% CI: 1.7-17.0, Table S7, Additional file 7). In addition, we found a 1.5-fold increase in the risk (95% CI: 0.6-3.6) of engaging in the following behaviors as an adult: smoking > 20 cigarettes a day, drinking 5+/4+ alcoholic beverages per drinking day, and using major drugs, rising to a 1.9-fold increased risk among subjects in the highest exposure tertile (95% CI: 0.6-5.7). Two-way combinations of these behaviors were rare and not associated with PCE exposure.

The magnitude of the main associations observed for smoking, drinking and drug use increased when the analysis was limited to subjects whose mothers, according to their self reports, did not smoke cigarettes or marijuana or drink alcoholic beverages during the subject's gestation (Table S8, Additional file 8). In particular, subjects in the highest exposure tertile had a 2.9-fold increased risk of frequent drinking as a teenager (95% CI: 1.3-6.1), and a 2.3-fold increased risk of using two or more major drugs as a teenager (95% CI: 1.3-4.1), stemming mainly from increases in the risk of using inhalants, crack/cocaine, psychedelics/hallucinogens, club/designer drugs, Ritalin with a prescription. These subjects also had a 2.1- fold increase in the risk of using two or more major drugs as an adult (95% CI: 1.3-3.5), due primarily to increases in the risk of using crack/cocaine, club/designer drugs, and Ritalin with a prescription.

## Discussion

The results presented here suggest that risky behaviors involving smoking, alcohol consumption and drug use are increased among individuals who were exposed to PCE-contaminated drinking water during gestation and early childhood. In particular, the most highly exposed subjects reported increases in the risk of using multiple major drugs both as a teenager and as an adult. Specific drugs for which elevated risks were observed included crack/cocaine, psychedelics/hallucinogens, club/designer drugs, Ritalin without a prescription, and heroin. Increases in the risk of certain smoking and drinking behaviors were also seen among the highest exposure group. Limiting the analyses to subjects without prenatal exposure to maternal cigarette smoking, marijuana use and alcohol consumption strengthened these associations.

Any causal interpretation of these findings should consider the following study limitations. First, the results are likely affected by exposure misclassification. Because historical exposure measurements were unavailable, we estimated PCE exposure during the study period using EPANET water distribution modeling software that was modified to incorporate a leaching and transport model [21,23]. The model was applied to water distribution system conditions in 1980 and was assumed to be representative of the entire exposure period. Furthermore, the exposure assessment predicted the annual mass of PCE delivered to each subject's residence during gestation and early childhood and, because information on maternal water use was available for only 58% of the study population, we did not incorporate these data into our analyses. Results from validation studies indicate reasonable correlation between our exposure estimates and PCE concentrations in historical water samples [31,32], but non-differential exposure misclassification likely biased the findings from dichotomous comparisons (e.g., any exposure vs. none) towards the null [33]. The expected direction of bias for comparisons involving the exposure tertiles is more difficult to predict, but associations seen among subjects in the highest tertile are likely to be attenuated while those in the middle category could have either an upward or downward bias.

Another limitation stems from the use of self-reports as the source of information on the risky behaviors. While the prevalence of these behaviors among study subjects is similar or higher than reported in independent surveys of Cape Cod and other Massachusetts residents [34,35], some faulty reporting was likely. However, since most subjects did not know their exposure status (see results section), inaccurate reporting was likely to be non-differential and so would not have affected the risk ratios from this cohort study [36].

Still another limitation stems from possible residual confounding by some contextual feature of highly exposed communities. In fact, 52% of highly exposed residents came from the town of Falmouth. However, there was no association between town of birth/residence and the prevalence of risky behaviors. For example, risk ratios for multiple major drug use as a teen and as an adult were 1.1 and 1.0, respectively, when Falmouth residents were compared to residents of the other study towns.

Residual confounding is also possible from missing data on several risk factors for substance use during childhood and adolescence, including parental smoking, alcohol and drug use, poor parental supervision, peer influences, behavior problems, and adverse events [37-41]. In order to account for the associations observed in this study, these factors would need to be highly correlated with PCE exposure, an unlikely scenario given the irregular pattern of the PCE contamination across the neighborhoods of Cape Cod. In fact, our prior studies of this cohort also found little or no confounding for the associations being investigated [42,43]. Furthermore, the magnitude of most associations increased when the analysis was restricted to subjects whose mothers reported that they did not smoke cigarettes or marijuana, or drink alcoholic beverages during the subject's gestation, suggesting that residual confounding by other similar factors could have led to a downward bias.

A further limitation stems from the study's low response rate. Although this problem reduced the statistical power of the study, the following evidence suggests that it did not result in selection bias. First, most available characteristics of participants and non-participants were similar, including initial PCE exposure status. Second, while participants were more likely to be female (60.7% of participants vs. 43.5% of non-participants), and have mothers with some college education (61.0% for participants vs. 49.3% for non-participants), these differences were equally present for exposed and unexposed non-participants. Third, losses stemming from the death of potential participants were small and unrelated to initial PCE exposure status (n = 111, Table 1). In addition, our review of death records from the Massachusetts Registry of Vital Records and Statistics and the National Death Index suggested that only four deaths were associated with substance use; two of these deaths occurred among exposed subjects and two occurred among unexposed subjects.

Intermediate factors that may have contributed to a subject's decision to engage in risky behaviors include early puberty [44], learning disabilities [45], and mental illness [46]. Analyses of our study population do not indicate any association between high PCE exposure and either early puberty or learning disabilities [16]. Analyses of the occurrence of mental illness in relation to early life exposure to PCE are currently underway.

PCE's potential to cause neurotoxic effects has been established through numerous animal and human studies [3]. Because of its small size and fat solubility, PCE readily crosses the blood brain barrier and has a high affinity for the lipophilic tissues of the central nervous system. Most epidemiologic studies of adults with occupational exposure to PCE and related solvents have reported impairments in cognition, memory, attention, and executive function [4-9]. Increases in anxiety and depression have also been reported in several of these studies [4,5,7,10,11].

As noted in the Introduction, studies of the neurotoxic effects among individuals with early life exposure to organic solvents have produced mixed results. Eskenazi et al. found no deficits in intellectual ability, motor skills or memory among pre-school children whose mothers had jobs involving solvent exposure during pregnancy [13]. In addition, no meaningful differences were seen in two studies of cognitive and behavioral function among children attending a nursery school and day care center who were exposed to PCE from nearby dry cleaning facilities [14,15]. Our prior cohort study on the reproductive and developmental effects of prenatal and early postnatal PCE exposure also did not observe any associations with disorders of attention, learning, and behavior throughout childhood [16]. In contrast, Till et al. found that pre-school children whose mothers were exposed to organic solvents during pregnancy had lower scores on language tests, reduced graphomotor skills, and more behavioral problems than unexposed children [17]. In addition, Laslo-Baker et al. found that preschool children with prenatal exposure to organic solvent mixtures scored lower on tests of general intelligence, language and motor skills [18]. The inconsistent findings among these studies may stem from differences in the sensitivity of measures used to assess deficits in brain function [16].

To the best of our knowledge, no prior study has investigated other lasting consequences of early life exposure to PCE and related solvents. However, because PCE and alcohol have similar neurotoxic effects [3], two long-term birth cohort studies of prenatal alcohol exposure provide pertinent data for comparison. A prospective cohort study from Seattle, Washington found a significant association between prenatal alcohol exposure and drinking problems among offspring at age 14 and 21 years, even after controlling for family history of alcohol problems, prenatal maternal smoking, prenatal and postnatal parental drug use, postnatal parental alcohol use, and parenting style [19,47]. Another prospective cohort study from Brisbane, Australia also found that prenatal alcohol exposure was associated with heavy alcohol consumption and alcohol disorders among offspring during adolescence and early adulthood [20,48]. In particular, investigators observed a 2.74-fold increase in the risk of consuming three or more drinks per drinking occasion at age 14 years among offspring whose mothers consumed at least three drinks per drinking occasion during pregnancy (95% CI:1.70-4.22) [48]. Additionally, they observed a 3.29-fold increased risk of developing alcohol disorders at ages 18-21 years among offspring whose mothers consumed at least three drinks per drinking occasion during early pregnancy (95% CI: 1.74-6.24) [20]. These investigators also adjusted for numerous confounding variables, including gender, maternal smoking, education, age, anxiety, depression, and child behavior. While results from these two studies may be still be confounded by genetic predisposition and unmeasured environmental influences for alcohol consumption, they are supported by animal data suggesting that early life exposure to alcohol increases subsequent affinity, either by creating a preference for similar stimuli or by developing an association between alcohol's odor and taste and its pharmacological effect [49].

The mode of action by which PCE and other organic solvents may cause neurological effects, including an affinity for risky behaviors, is currently unknown [3]. There is evidence to support neurotoxic mechanisms involving the peroxidation of cell membrane lipids [50], changes in the fatty acid profile of the brain [51], and loss of myelin [52]. In addition, experiments suggest that the neurotoxic mechanism may involve ligand-gated ion channel activity via the following receptors: GABA_A_, glycine, NMDA, glutamate kainite, and AMPA [53-57]. In fact, it has been suggested that exposure to agents such as ethanol during synaptogenesis can trigger substantial apoptotic neurodegeneration because these agents interfere with the action of neurotransmitters and GABA_A _receptors [58].

In summary, the results of this study suggest that risky behaviors, particularly drug use, are increased among adults exposed to high levels of PCE during gestation and early childhood. Because this study has several limitations and is the first to report this association, these findings should be confirmed in follow-up investigations of similarly exposed populations. Because PCE remains a commonly used commercial solvent that exposes workers and consumers and causes frequent contamination of drinking water from groundwater sources, it is important to determine its long-term impact on behavior.

## Conclusions

Numerous studies of adults with acute and chronic solvent exposure have shown adverse impacts on cognition, executive function, behavior and mood. However, no prior study has investigated the long-term impact of prenatal and early childhood exposure to the prevalent organic solvent tetrachloroethylene (PCE) on the development of risky behaviors. We undertook a retrospective cohort study to examine whether early life exposure to PCE-contaminated drinking water influenced the adoption of cigarette smoking, alcohol consumption, and drug use among adults from Cape Cod, Massachusetts. The results suggest that these risky behaviors, particularly drug use, are increased among adults with PCE exposure levels during gestation and early childhood. Specific illicit drugs for which increased risks were observed included crack/cocaine, pychedelics/hallucinogens, club/designer drugs, Ritalin without a prescription, and heroin. Limiting the analyses to subjects without prenatal exposure to maternal cigarette smoking, marijuana use and alcohol consumption strengthened these associations. Because this study has many limitations and is the first to report this association, additional long-term studies of similarly exposed populations are needed.

## Abbreviations

CI: Confidence Interval; DEP: Department of Environmental Protection; GEE: Generalized estimating equation; IRB: Institutional Review Board; PCE: Tetrachloroethylene; RDD: Relative delivered dose; RR: Risk Ratio; SNARL: Suggested no adverse response action level; VL/AC: vinyl-lined asbestos-cement.

## Competing interests

Dr. David Ozonoff is Co-editor-in-Chief of *Environmental Health*. He has recused himself from all decisions involving the acceptance and publication of this manuscript. At the request of the Commonwealth of Massachusetts, in 1980 Dr. Ozonoff was a witness in bankruptcy court in a suit against the Johns-Manville Corporation, manufacturers of the ACVL water mains. He has also, on occasion, testified in personal injury and property damage cases involving exposure to tetrachloroethylene and trichloroethylene. Three years ago, Dr. Aschengrau served as a consultant in a personal injury case involving chlorinated solvent contamination. None of the parties in any litigation supported, reviewed or had knowledge of this paper. None of the other authors of this study have any competing interests.

## Authors' contributions

AA conceived the study and its design, coordinated data collection and analysis, and drafted the initial manuscript. JW, PAJ, TW, and VV provided technical input to data collection, exposure assessment, data analysis, and manuscript preparation. MER and LG conducted the data collection, geocoding, and exposure assessments. MRW and BRM participated in the data collection and conducted the data analyses. DO and RFW provided technical input to the study design, data collection, and manuscript preparation. All authors read and approved the final manuscript.

## Supplementary Material

Additional file 1 **Table S1 Distribution of Selected Characteristics of Subjects and Parents by PCE Exposure Status**. Click here for file

Additional file 2 **Table S2 Distribution of Cumulative PCE Exposure (in grams) among Exposed Subjects**. Click here for file

Additional file 3 **Table S3 Prenatal and Early Childhood Exposure to Tetrachloroethylene and the Risk of Cigarette Smoking**. Click here for file

Additional file 4 **Table S4 Prenatal and Early Childhood Exposure to Tetrachloroethylene and the Risk of Alcohol Use**. Click here for file

Additional file 5 **Table S5 Prenatal and Early Childhood Exposure to Tetrachloroethylene and the Risk of Teenage Drug Use**. Click here for file

Additional file 6 **Table S6 Prenatal and Early Childhood Exposure to Tetrachloroethylene and the Risk of Adult Drug Use**. Click here for file

Additional file 7 **Table S7 Prenatal and Early Childhood Exposure to Tetrachloroethylene and Multiple Risky Behaviors**. Click here for file

Additional file 8 **Table S8 Prenatal and Early Childhood Exposure to Tetrachloroethylene and Selected Risky Behaviors Among Subjects Without a History of Prenatal Exposure to Cigarette Smoke, Marijuana or Alcohol**. Click here for file
