# Supplementary material for: Affinity for risky behaviors following prenatal and early childhood exposure to tetrachloroethylene (PCE)-contaminated drinking water: a retrospective cohort study
Source: Environ Health. 2011 Dec 2;10:102. doi: 10.1186/1476-069X-10-102 (PMC3268745; doi:10.1186/1476-069X-10-102)
Supplement: Additional file 1 — Table S1 Distribution of Selected Characteristics of Subjects and Parents by PCE Exposure Status. [file 1476-069X-10-102-S1.DOCX]

| Table S1 Distribution of Selected Characteristics of Subjects and Parents by PCE Exposure Status | | | | |
| --- | --- | --- | --- | --- |
| Characteristic | Prenatal and Early Childhood Exposure  (N=831) n % | | Unexposed  (N=547)  n % | |
| Year of birth |  |  |  |  |
| 1969-1974 | 166 | 20.0 | 131 | 23.9 |
| 1975-1980 | 435 | 52.3 | 288 | 52.7 |
| 1981-1983 | 230 | 27.7 | 128 | 23.4 |
| Current age  (n, mean, sd) | 831 | 29.2 (3.6) | 547 | 29.6 (3.8) |
| Gender |  |  |  |  |
| Male | 331 | 39.8 | 216 | 39.5 |
| Female | 500 | 60.2 | 331 | 60.5 |
| % White race | 818 | 98.4 | 539 | 98.5 |
| Current Educational Level |  |  |  |  |
| High school graduate or less | 128 | 15.4 | 67 | 12.2 |
| Some college | 192 | 23.1 | 144 | 26.3 |
| Four year college grad or  higher | 510 | 61.4 | 335 | 61.2 |
| Missing | 1 | 0.1 | 1 | 0.2 |
| Currently Employed |  |  |  |  |
| Yes | 719 | 86.5 | 487 | 89.0 |
| No | 92 | 11.1 | 54 | 9.9 |
| Missing | 20 | 2.4 | 6 | 1.1 |
| Current marital status |  |  |  |  |
| Single | 272 | 32.7 | 157 | 28.7 |
| Married or cohabitating | 536 | 64.5 | 371 | 67.8 |
| Other | 19 | 2.3 | 12 | 2.2 |
| Missing | 4 | 0.5 | 7 | 1.3 |
| Ever had solvent-exposed job |  |  |  |  |
| Yes | 123 | 14.8 | 71 | 13.0 |
| No | 687 | 82.7 | 461 | 84.3 |
| Missing | 21 | 2.5 | 15 | 2.7 |
| Ever had solvent-exposed hobby |  |  |  |  |
| Yes | 700 | 84.2 | 462 | 84.5 |
| No | 124 | 14.9 | 79 | 14.4 |
| Missing | 7 | 0.8 | 6 | 1.1 |
| History of learning problem |  |  |  |  |
| Yes | 195 | 23.5 | 126 | 23.0 |
| No | 624 | 75.1 | 415 | 75.9 |
| Missing | 12 | 1.4 | 6 | 1.1 |
| History of repeating a grade |  |  |  |  |
| Yes | 97 | 11.7 | 75 | 13.7 |
| No | 725 | 87.2 | 468 | 85.6 |
| Missing | 9 | 1.1 | 4 | 0.7 |
| History of mental disorder |  |  |  |  |
| Yes | 203 | 24.4 | 117 | 21.4 |
| No | 617 | 74.2 | 426 | 77.9 |
| Missing | 11 | 1.3 | 4 | 0.7 |
| Mother’s age at subject’s birth  (n, mean (sd)) | 831 | 27.2 (4.7) | 547 | 27.5 (4.4) |
| Father’s age at subject’s birth  (n, mean (sd)) | 831 | 29.8 (5.7) | 547 | 29.8 (5.3) |
| Mother’s educational level at subject’s birth |  |  |  |  |
| High school graduate or less | 327 | 39.4 | 178 | 32.5 |
| Some college | 243 | 29.2 | 188 | 34.4 |
| Four year college grad or  higher | 260 | 31.3 | 180 | 32.9 |
| Missing | 1 | 0.1 | 1 | 0.2 |
| Father’s occupation at subject’s birth |  |  |  |  |
| White collar | 420 | 50.5 | 257 | 47.0 |
| Blue collar | 275 | 33.1 | 170 | 31.1 |
| Other | 126 | 15.2 | 112 | 20.5 |
| Missing | 10 | 1.2 | 8 | 1.5 |
| Mother received prenatal care during subject’s gestation |  |  |  |  |
| Yes | 794 | 95.5 | 520 | 95.1 |
| No | 4 | 0.5 | 0 | 0.0 |
| Missing | 33 | 4.0 | 27 | 4.9 |
| Mother’s cigarette smoking during subject’s gestation |  |  |  |  |
| 11+ cigarettes a day | 108 | 13.0 | 59 | 10.8 |
| 10 or fewer cigarettes a day | 74 | 8.9 | 54 | 9.9 |
| None | 483 | 58.1 | 330 | 60.3 |
| Missing | 166 | 20.0 | 104 | 19.0 |
| Mother’s alcohol consumption during subject’s gestation |  |  |  |  |
| 1+ drinks a week | 109 | 13.1 | 76 | 13.9 |
| 1-3 drinks a month | 193 | 23.2 | 125 | 22.9 |
| None | 361 | 43.4 | 242 | 44.2 |
| Missing | 168 | 20.2 | 104 | 19.0 |
| Mother’s use of marijuana during subject’s gestation |  |  |  |  |
| Yes | 25 | 3.0 | 18 | 3.3 |
| No | 640 | 77.0 | 420 | 76.8 |
| Missing | 166 | 20.0 | 109 | 19.9 |
| Mother’s medical and obstetrical complications during subject’s gestation |  |  |  |  |
| Yes | 122 | 14.7 | 108 | 19.7 |
| No | 536 | 64.5 | 331 | 60.5 |
| Missing | 173 | 20.8 | 108 | 19.7 |
| Mother’s occupational exposure to solvents |  |  |  |  |
| Yes | 76 | 9.1 | 51 | 9.3 |
| No | 573 | 69.0 | 381 | 69.7 |
| Missing | 182 | 21.9 | 115 | 21.0 |
| Subject’s birth weight (n, mean, sd) | 823 | 3,443 (506) | 499 | 3,414 (534) |
| Subject’s gestational age (n, mean, sd) | 790 | 40.1 (2.5) | 516 | 39.9 (2.4) |
| Multiple Pregnancy | 21 | 2.5 | 20 | 3.7 |
| Subject breast fed |  |  |  |  |
| Yes | 406 | 48.9 | 299 | 54.7 |
| No | 254 | 30.6 | 140 | 25.6 |
| Missing | 171 | 20.6 | 108 | 19.7 |
| Number of older siblings |  |  |  |  |
| 0 | 350 | 42.1 | 262 | 47.9 |
| 1 | 287 | 34.5 | 163 | 29.8 |
| 2+ | 193 | 23.2 | 119 | 21.8 |
| Missing | 1 | 0.1 | 3 | 0.5 |
| Sibling died | 16 | 1.9 | 15 | 2.7 |
| Parent(s) divorced, separated, or died after subject’s birth | 51 | 6.1 | 32 | 5.9 |
| Family history of mental disorder |  |  |  |  |
| Yes | 332 | 40.0 | 219 | 40.0 |
| No | 444 | 53.4 | 303 | 55.4 |
| Missing | 55 | 6.6 | 25 | 4.6 |
