# Supplementary material for: Affinity for risky behaviors following prenatal and early childhood exposure to tetrachloroethylene (PCE)-contaminated drinking water: a retrospective cohort study
Source: Environ Health. 2011 Dec 2;10:102. doi: 10.1186/1476-069X-10-102 (PMC3268745; doi:10.1186/1476-069X-10-102)
Supplement: Additional file 2 — Table S2 Distribution of Cumulative PCE Exposure (in grams) among Exposed Subjects. [file 1476-069X-10-102-S2.DOCX]

| Table S2 Distribution of Cumulative PCE Exposure (in grams) among Exposed Subjects (N=831) | | | |
| --- | --- | --- | --- |
|  | Prenatal Exposure | Early Childhood  Exposure | Prenatal and Early Childhood Exposure Combined |
| Minimum | 9.092E-04 | 8.448E-03 | 1.108E-02 |
| 10^th^ Percentile | 0.4 | 1.2 | 1.9 |
| 33^rd^ Percentile | 1.9 | 5.3 | 7.7 |
| 50^th^ Percentile | 7.3 | 24.0 | 34.5 |
| 67^th^ Percentile | 18.1 | 56.9 | 77.6 |
| 90^th^ Percentile | 78.1 | 245.7 | 342.7 |
| Maximum | 1,255.2 | 3,812.9 | 4,667.9 |
| Mean (SD) | 32.6 (88.6) | 109.0 (283.3) | 141.6 (358.1) |
