# Supplementary material for: Affinity for risky behaviors following prenatal and early childhood exposure to tetrachloroethylene (PCE)-contaminated drinking water: a retrospective cohort study
Source: Environ Health. 2011 Dec 2;10:102. doi: 10.1186/1476-069X-10-102 (PMC3268745; doi:10.1186/1476-069X-10-102)
Supplement: Additional file 3 — Table S3 Prenatal and Early Childhood Exposure to Tetrachloroethylene and the Risk of Cigarette Smoking. [file 1476-069X-10-102-S3.DOC]

Table S3 Prenatal and Early Childhood Exposure to Tetrachloroethylene and the Risk of Cigarette Smoking

Crude Simple GEE

Outcome Exposure % Yes (n/N) RR (95% CI) RR (95% CI)

Category/

Percentile

Ever smoked regularly vs. Never smoked regularly ^1^ Any 36.9 (303/821) 1.0 (0.9-1.2) 1.0 (0.9-1.2)

>=67^th^ 45.0 (122/271) 1.3 (1.1-1.5) 1.2 (1.0-1.5)

33^rd^- <67^th^ 29.1 (81/278) 0.8 (0.7-1.0) 0.8 (0.7-1.0)

>0- <33^rd^  36.8 (100/272) 1.0 (0.9-1.3) 1.0 (0.9-1.3)

None 35.2 (191/543) Reference Reference

Started smoking at <=13 years vs. Never smoked regularly^2^ Any 7.2 (40/558) 1.0 (0.6-1.7) 1.0 (0.6-1.6)

>=67^th^ 11.8 (20/169) 1.7 (1.0-3.0) 1.4 (0.8-2.5)

33^rd^- <67^th^ 3.9 (8/205) 0.6 (0.3-1.2) 0.6 (0.3-1.3)

>0-<33^rd^ 6.5 (12/184) 0.9 (0.5-1.8) 1.0 (0.5-1.8)

None 6.9 (26/378) Reference Reference

Smoked 20+ cigarettes a day vs. Never smoked regularly^3^ Any 12.6 (75/593) 1.1 (0.8-1.5) 1.1 (0.8-1.5)

>=67^th^ 15.8 (28/177) 1.3 (0.9-2.0) 1.3 (0.9-2.0)

33^rd^- <67^th^ 12.1 (27/224) 1.0 (0.6-1.6) 1.0 (0.7-1.6)

>0-<33^rd^ 10.4 (20/192) 0.9 (0.5-1.4) 0.9 (0.5-1.4)

None 12.0 (48/400) Reference Reference

1 Comparison excludes subjects who smoked 100+ cigarettes but never became regular smokers

2 Comparison excludes subjects who started smoking at age 14 and older

3 Comparison excludes subjects who smoked < 20 cigarettes a day
