# Supplementary material for: Affinity for risky behaviors following prenatal and early childhood exposure to tetrachloroethylene (PCE)-contaminated drinking water: a retrospective cohort study
Source: Environ Health. 2011 Dec 2;10:102. doi: 10.1186/1476-069X-10-102 (PMC3268745; doi:10.1186/1476-069X-10-102)
Supplement: Additional file 4 — Table S4 Prenatal and Early Childhood Exposure to Tetrachloroethylene and the Risk of Alcohol Use. [file 1476-069X-10-102-S4.DOC]

Table S4 Prenatal and Early Childhood Exposure to Tetrachloroethylene and the Risk of Teenage and Adult Alcohol Use

Crude Simple GEE

Outcome Exposure % Yes (n/N) RR (95% CI) RR (95% CI)

Category/

Percentile

First drank at <=13 years vs. 14+ years^1^ Any 19.6 (159/811) 1.1 (0.8-1.3) 1.1 (0.8-1.3)

>67^th^ 21.9 (59/269) 1.2 (0.9-1.6) 1.2 (0.9-1.6)

33^rd^- <67^th^  18.4 (51/277) 1.0 (0.7-1.3) 1.0 (0.7-1.4)

>0-<33^rd^ 18.5 (49/265) 1.0 (0.7-1.4) 1.0 (0.7-1.4)

None 18.6 (98/528) Reference Reference

Drank >8 days/mo as teen vs. Never drank as a teen^2^ Any 26.2 (68/260) 1.1 (0.8-1.5) 1.1 (0.8-1.5)

>67^th^ 38.8 (33/85) 1.6 (1.1-2.4) 1.6 (1.1-2.3)

33^rd^ - <67^th^  19.8 (18/91) 0.8 (0.4-1.5) 0.8 (0.5-1.3)

>0-<33^rd^ 20.2 (17/84) 0.9 (0.5-1.4) 0.8 (0.5-1.4)

None 23.7 (42/177) Reference Reference

Drank >=5/4 drinks/drinking day as teen Any 59.9 (2 87/479) 1.1 (1.0-1.2) 1.1 (0.9-1.2)

vs. Never drank as a teen^3^ >67^th^ 64.1 (93/145) 1.2 (1.0-1.4) 1.1 (1.0-1.3)

33^rd^ - <67^th^ 55.8 (92/165) 1.0 (0.8-1.2) 1.0 (0.8-1.2)

>0-<33^rd^ 60.4 (102/169) 1.1 (0.9-1.3) 1.1 (0.9-1.3)

None 55.4 (168/303) Reference Reference

Drank >8 days/ in past 30 days Any 63.2 (256/ 405) 1.0 (0.9-1.1) 1.0 (0.9-1.1)

vs. Did not drink in past 30 days^4^ >67^th^ 69.3 (88/127) 1.1 (1.0-1.3) 1.1 (1.0-1.3)

33^rd^ - <67^th^  59.5 (75/126) 1.0 (0.8-1.1) 0.9 (0.8-1.1)

>0-<33^rd^ 61.2 (93/152) 1.0 (0.8-1.1) 1.0 (0.8-1.1)

None 62.6 (181/289 ) Reference Reference

Drank >=5/4 drinks/drinking day in past 30 days Any 47.2 (133/282) 1.1 (0.9-1.3) 1.1 (0.9-1.3)

vs. Did not drink in past 30 days^5^ >67^th^ 57.6 (53/92) 1.3 (1.0-1.7) 1.3 (1.0-1.7)

33^rd^ - <67^th^  41.4 (36/87) 1.0 ((0.7-1.3) 0.9 (0.7-1.3)

>0-<33^rd^ 42.7 (44/103) 1.0 (0.7-1.3) 1.0 (0.7-1.3)

None 43.5 (83/191) Reference Reference

Table S4 Prenatal and Early Childhood Exposure to Tetrachloroethylene and the Risk of Alcohol Use

1 Comparison excludes subjects who never drank as a teen

2 Comparison excludes subjects who drank <= 8 days/month as a teen

3 Comparison excludes subjects who drank <5/4 drinks/drinking day as a teen

4 Comparison excludes subjects who drank <= 8 days/month in past 30 days

5 Comparison excludes subjects who drank <5/4 drinks/drinking day in past 30 days
