# Supplementary material for: Affinity for risky behaviors following prenatal and early childhood exposure to tetrachloroethylene (PCE)-contaminated drinking water: a retrospective cohort study
Source: Environ Health. 2011 Dec 2;10:102. doi: 10.1186/1476-069X-10-102 (PMC3268745; doi:10.1186/1476-069X-10-102)
Supplement: Additional file 5 — Table S5 Prenatal and Early Childhood Exposure to Tetrachloroethylene and the Risk of Teenage Drug Use. [file 1476-069X-10-102-S5.DOC]

Table S5 Prenatal and Early Childhood Exposure to Tetrachloroethylene and the Risk of Teenage Drug Use

Crude Simple GEE

Outcome Exposure % Yes (n/N) RR (95% CI) RR (95% CI)

Category/

Percentile

Any drugs vs. Never used any drugs^1^ Any 67.3 (459/682) 1.0 (1.0-1.1) 1.0 (1.0-1.1)

>67^th^ 74.5 (172/231) 1.2 (1.0-1.3) 1.2 (1.1-1.3)

33^rd^ - <67^th^  60.5 (138/228) 0.9 (0.8-1.1) 0.9 (0.8-1.1)

0-<33^rd^ 66.8 (149/223) 1.0 (0.9-1.2) 1.0 (0.9-1.2)

None 64.1 (293/457) Reference Reference

2+ Drugs vs. Never used any drugs ^1,2^ Any 49.1 (215/438) 1.2 (1.0-1.4) 1.2 (1.0-1.4)

>67^th^ 56.9 (78/137) 1.4 (1.1-1.7) 1.4 (1.1-1.7)

33^rd^ - <67^th^  41.6 (64/154) 1.0 (0.8-1.3) 1.0 (0.8-1.3)

>0-<33^rd^ 49.7 (73/147) 1.2 (1.0-1.5) 1.2 (0.9-1.5)

None 41.6 (117/281) Reference Reference

Any major drugs vs. Never used any drugs^1,3^ Any 50.1 (224/447) 1.2 (1.0-1.4) 1.2 (1.0-1.4)

>67^th^ 58.7 (84/143) 1.4 (1.2-1.7) 1.4 (1.1-1.7)

33^rd^ - <67^th^  42.7 (67/157) 1.0 (0.8-1.3) 1.0 (0.8-1.3)

0-<33^rd^ 49.7 (73/147) 1.2 (1.0-1.5) 1.2 (0.9-1.5)

None 42.0 (119/283) Reference Reference

2+ Major drugs vs. Never used any drugs^1,4^ Any 35.7 (124/347) 1.4 (1.0-1.8) 1.4 (1.0-1.8)

>67^th^ 43.3 (45/104) 1.6 (1.2-2.2) 1.6 (1.2-2.2)

33^rd^ - <67^th^  30.8 (40/130) 1.2 (0.8-1.6) 1.2 (0.8-1.6)

>0-<33^rd^ 34.5 (39/113) 1.3 (0.9-1.8) 1.3 (0.9-1.8)

None 26.5 (59/223) Reference Reference

Marijuana vs. Never used any drugs^1,5^ Any 66.8 (449/672) 1.0 (1.0-1.1) 1.0 (1.0-1.1)

>67^th^ 73.7 (165/224) 1.2 (1.0-1.3) 1.2 (1.0-1.3)

33^rd^- <67^th^  60.0 (135/225) 0.9 (0.8-1.1) 0.9 (0.8-1.1)

>0-<33^rd^ 66.8 (149/223) 1.0 (0.9-1.2) 1.0 (0.9-1.2)

None 64.0 (291/455) Reference Reference

Table S5 Prenatal and Early Childhood Exposure to Tetrachloroethylene and the Risk of Teenage Drug Use

Crude Simple GEE

Outcome Exposure % Yes (n/N) RR (95% CI) RR (95% CI)

Category/

Percentile

Inhalants vs. Never used any drugs^1,5^  Any 18.6 (51/274) 1.1 (0.7-1.6) 1.1 (0.7-1.6)

>67^th^ 21.3 (16/75) 1.2 (0.7-2.1) 1.2 (0.7-2.1)

33^rd^ - <67^th^  16.7 (18/108) 0.9 (0.6-1.6) 0.9 (0.6-1.6)

>0-<33^rd^ 18.7 (17/91) 1.1 (0.6-1.8) 1.1 (0.6-1.8)

None 17.6 (35/199) Reference Reference

Crack/cocaine vs. Never used any drugs^1,5^ Any 29.2 (92/315) 1.6 (1.1-2.2) 1.6 (1.1-2.2)

>67^th^ 38.5 (37/96) 2.1 (1.4-3.1) 2.1 (1.4-3.0)

33^rd^ - <67^th^  22.4 (26/116) 1.2 (0.8-1.9) 1.2 (0.8-1.9)

>0-<33^rd^ 28.2 (29/103) 1.5 (1.0-2.3) 1.5 (0.9-2.3)

None 18.4 (37/201) Reference Reference

Psychedelics/Hallucinogens vs. Never used any drugs^1,5^ Any 41.8 (160/383) 1.2 (1.0-.1.5) 1.2 (1.0-1.5)

>67^th^ 47.3 (53/112) 1.4 (1.1-1.8) 1.4 (1.1-1.8)

33^rd^ - <67^th^  36.6 (52/142) 1.1 (0.8-1.4) 1.1 (0.8-1.4)

0-<33^rd^ 42.6 (55/129) 1.2 (1.0-1.6) 1.3 (1.0-1.6)

None 34.1 (85/249) Reference Reference

Club/Designer Drugs vs. Never used any drugs^1,5^ Any 27.8 (86/309) 1.4 (1.0-2.0) 1.5 (1.0-2.1)

>67^th^ 39.8 (39/98) 2.1 (1.4-3.0) 2.1 (1.5-3.1)

33^rd^ - <67^th^  16.7 (18/108) 0.9 (0.5-1.4) 0.9 (0.5-1.5)

>0-<33^rd^ 28.2 (29/103) 1.5 (1.0-2.2) 1.5 (0.9-2.3)

None 19.2 (39/203) Reference Reference

Ritalin without a prescription vs. Never used any drugs^1,5^ Any 23.6 (69/292) 1.5 (1.0-2.3) 1.5 (1.0-2.2)

>67^th^ 33.7 (30/89) 2.2 (1.4-3.4) 2.1 (1.4-3.3)

33^rd^ - <67^th^  16.7 (18/108) 1.1 (0.6-1.8) 1.1 (0.6-1.8)

>0-<33^rd^ 22.1 (21/95) 1.4 (0.9-2.4) 1.3 (0.8-2.2)

None 15.5 (30/194) Reference Reference

Table S5 Prenatal and Early Childhood Exposure to Tetrachloroethylene and the Risk of Teenage Drug Use

Crude Simple GEE

Outcome Exposure % Yes (n/N) RR (95% CI) RR (95% CI)

Category/

Percentile

Heroin vs. Never used any drugs^1,5^ Any 2.6 (6/229) 0.9 (0.3-2.9) 0.9 (0.3-2.9)

>67^th^ 6.3 (4/63) 2.1 (0.6-7.7) 2.1 (0.6-7.7)

33^rd^ - <67^th^  1.1 (1/91) 0.4 (0.0-3.1) 0.4 (0.0-3.1)

>0-<33^rd^ 1.3 (1/75) 0.5 (0.1-3.8) 0.5 (0.1-3.8)

None 3.0 (5/169) Reference Reference

1 Referent group is comprised of subjects who never used drugs as a teen or an adult

2 Comparison excludes subjects who used only one drug as a teen

3 Comparison excludes subjects who used only marijuana as a teen

4 Comparison excludes subjects who used only marijuana or one major drug as a teen

5 Comparison excludes subjects who used any other type of drug as a teen
