# Supplementary material for: Affinity for risky behaviors following prenatal and early childhood exposure to tetrachloroethylene (PCE)-contaminated drinking water: a retrospective cohort study
Source: Environ Health. 2011 Dec 2;10:102. doi: 10.1186/1476-069X-10-102 (PMC3268745; doi:10.1186/1476-069X-10-102)
Supplement: Additional file 6 — Table S6 Prenatal and Early Childhood Exposure to Tetrachloroethylene and the Risk of Adult Drug Use. [file 1476-069X-10-102-S6.DOC]

Table S6 Prenatal and Early Childhood Exposure to Tetrachloroethylene and the Risk of Adult Drug Use

Crude Simple GEE

Outcome Exposure % Yes (n/N) RR (95% CI) RR (95% CI)

Category/

Percentile

Any drugs vs. Never used any drugs^1^ Any 70.4 (531/754) 1.0 (1.0-1.1) 1.0 (1.0-1.1)

>67^th^ 76.2 (189/248 ) 1.1 (1.0-1.2) 1.1 (1.0-1.2)

33^rd^ - <67^th^  64.0 (160/250 ) 0.9 (0.8-1.1) 1.0 (0.9-1.1)

0-<33^rd^ 71.1 (182/256 ) 1.1 (1.0-1.2) 1.1 (1.0-1.2)

None 67.4 (339/503 ) Reference Reference

2+ Drugs vs. Never used any drugs^1,2^ Any 54.9 (271/494) 1.2 (1.0-1.4) 1.2 (1.0-1.4)

>67^th^ 63.1 (101/160 ) 1.4 (1.1-1.6) 1.4 (1.1-1.6)

33^rd^ - <67^th^  46.4 (78/168) 1.0 (0.8-1.2) 1.0 (0.8-1.2)

>0-<33^rd^ 55.4 (92/166) 1.2 (1.0-1.4) 1.2 (1.0-1.4)

None 46.6 (143/307) Reference Reference

Major drugs vs. Never used any drugs^1,3^  Any 56.4 (289/512) 1.1 (1.0-1.3) 1.1 (1.0-1.3)

>67^th^ 64.7 (108/167) 1.3 (1.1-1.5) 1.3 (1.1-1.5)

33^rd^ - <67^th^  48.0 (83/173) 1.0 (0.8-1.2) 1.0 (0.8-1.2)

0-<33^rd^ 57.0 (98/174) 1.1 (1.0-1.4) 1.1 (1.0-1.4)

None 49.7 (162/326) Reference Reference

2+ Major drugs vs. Never used any drugs^1,4^ Any 44.5 (179/402) 1.3 (1.0-1.6) 1.3 (1.0-1.6)

>67^th^ 53.9 (69/128) 1.6 (1.2-2.0) 1.5 (1.2-1.9)

33^rd^ - <67^th^  37.1 (53/143) 1.1 (0.8-1.4) 1.1 (0.8-1.4)

>0-<33^rd^ 43.5 (57/131) 1.3 (1.0-1.6) 1.2 (1.0-1.6)

None 34.7 (87/251) Reference Reference

Marijuana vs. Never used any drugs^1,5^ Any 69.6 (511/734) 1.1 (1.0-1.1) 1.1 (1.0-1.1)

>67^th^ 75.3 (180/239) 1.1 (1.0-1.3) 1.1 (1.0-1.3)

33^rd^- <67^th^  63.1 (154/244) 1.0 (0.9-1.1) 1.0 (0.9-1.1)

>0-<33^rd^ 70.5 (177/251) 1.1 (1.0-1.2) 1.1 (1.0-1.2)

None 66.0 (319/483) Reference Reference

Table S6 Prenatal and Early Childhood Exposure to Tetrachloroethylene and the Risk of Adult Drug Use

Crude Simple GEE

Outcome Exposure % Yes (n/N) RR (95% CI) RR (95% CI)

Category/

Percentile

Inhalants vs. Never used any drugs^1,5^ Any 6.3 (15/238) 1.1 (0.5-2.4) 1.1 (0.5-2.4)

>67^th^ 6.3 (4/63) 1.1 (0.4-3.4) 1.1 (0.4-3.4)

33^rd^ - <67^th^  6.3 (6/96) 1.1 (0.4-2.9) 1.1 (0.4-2.9)

>0-<33^rd^ 6.3 (5/79) 1.1 (0.4-3.1) 1.1 (0.4-3.1)

None 5.7 (10/174) Reference Reference

Crack/cocaine vs. Never used any drugs^1,5^  Any 43.5 (172/395) 1.2 (1.0-1.5) 1.2 (1.0-1.5)

>67^th^ 53.2 (67/126) 1.5 (1.2-1.9) 1.4 (1.1-1.8)

33^rd^ - <67^th^  34.3 (47/137) 0.9 (0.7-1.3) 0.9 (0.7-1.2)

>0-<33^rd^ 43.9 (58/132) 1.2 (0.9-1.6) 1.2 (0.9-1.6)

None 36.2 (93/257) Reference Reference

Psychedelics/Hallucinogens vs. Never used any drugs^1,5^ Any 42.1 (162/385) 1.2 (1.0-1.5) 1.2 (1.0-1.5)

>67^th^ 48.7 (56/115) 1.4 (1.1-1.8) 1.4 (1.1-1.8)

33^rd^ - <67^th^  33.8 (46/136) 1.0 (0.7-1.3) 1.0 (0.7-1.3)

0-<33^rd^ 44.8 (60/134) 1.3 (1.0-1.7) 1.3 (1.0-1.7)

None 34.7 (87/251) Reference Reference

Club/Designer Drugs vs. Never used any drugs^1,5^ Any 45.1 (183/406) 1.3 (1.1-1.6) 1.3 (1.1-1.6)

>67^th^ 52.0 (64/123) 1.5 (1.2-1.9) 1.5 (1.2-1.9)

33^rd^ - <67^th^  38.8 (57/147) 1.1 (0.9-1.5) 1.1 (0.9-1.5)

>0-<33^rd^ 45.6 (62/136) 1.3 (1.0-1.7) 1.3 (1.0-1.7)

None 34.4 (86/250) Reference Reference

Ritalin without a prescription vs. Never used any drugs^1,5^ Any 28.5 (89/312) 1.4 (1.0-2.0) 1.4 (1.0-2.0)

>67^th^ 38.5 (37/96) 1.9 (1.3-2.8) 1.9 (1.3-2.8)

33^rd^ - <67^th^  19.6 (22/112) 1.0 (0.6-1.6) 1.0 (0.6-1.5)

>0-<33^rd^ 28.8 (30/104) 1.4 (1.0-2.2) 1.4 (0.9-2.1)

None 20.0 (41/205) Reference Reference

Table S6 Prenatal and Early Childhood Exposure to Tetrachloroethylene and the Risk of Adult Drug Use

Crude Simple GEE

Outcome Exposure % Yes (n/N) RR (95% CI) RR (95% CI)

Category/

Percentile

Heroin vs. Never used any drugs^1,5^ Any 9.0 (22/245) 1.3 (0.7-2.6) 1.3 (0.7-2.6)

>67^th^ 11.9 (8/67) 1.8 (0.7-4.1) 1.7 (0.7-4.1)

33^rd^ - <67^th^  5.3 (5/95) 0.8 (0.3-2.1) 0.8 (0.3-2.1)

>0-<33^rd^ 10.8 (9/83) 1.6 (0.7-3.6) 1.6 (0.7-3.6)

None 6.8 (12/176) Reference Reference

1 Referent group is comprised of subjects who never used drugs as a teen or an adult

2 Comparison excludes subjects who used only one drug as an adult

3 Comparison excludes subjects who used only marijuana as an adult

4 Comparison excludes subjects who used only marijuana or one major drug as an adult

5 Comparison excludes subjects who used any other type of drug as an adult
