# Supplementary material for: Affinity for risky behaviors following prenatal and early childhood exposure to tetrachloroethylene (PCE)-contaminated drinking water: a retrospective cohort study
Source: Environ Health. 2011 Dec 2;10:102. doi: 10.1186/1476-069X-10-102 (PMC3268745; doi:10.1186/1476-069X-10-102)
Supplement: Additional file 7 — Table S7 Prenatal and Early Childhood Exposure to Tetrachloroethylene and Multiple Risky Behaviors. [file 1476-069X-10-102-S7.DOC]

Table S7 Prenatal and Early Childhood Exposure to Tetrachloroethylene and Multiple Risky Behaviors

Exposure % Yes (n/N) Crude RR (95% CI) Simple GEE RR (95% CI)

Multiple Teen Behaviors

Initiated smoking < 14 years, and

Drank alcoholic beverages >8 days/mo, and

Used major drugs Any 2.8 (16/569) 2.7 (0.9-8.1) 2.7 (0.9-8.0)

>=67^th^ 5.6 (10/180) 5.4 (1.7-17.0) 5.4 (1.7-17.0)

33^rd^- <67^th^ 1.0 (2/198) 1.0 (0.2-5.3) 1.0 (0.2-5.3)

>0- <33^rd^  2.1 (4/191) 2.1 (0.5-8.0) 2.0 (0.5-8.0)

None 1.0 (4/387) Reference Reference

Multiple Adult Behaviors

Smoked >20 cigarettes/day, and

Drank >=5/4 drinks/day, and

Used major drugs Any 3.2 (14/444) 1.5 (0.6-3.6) 1.5 (0.6-3.6)

>=67^th^ 4.0 (5/126) 1.9 (0.6-5.7) 1.9 (0.6-5.7)

33^rd^- <67^th^ 4.1 (7/171) 1.9 (0.7-5.4) 1.9 (0.7-5.4)

>0-<33^rd^ 1.4 (2/147) 0.6 (0.1-3.0) 0.6 (0.1-3.0)

None 2.1 (7/327) Reference Reference
