# Supplementary material for: Affinity for risky behaviors following prenatal and early childhood exposure to tetrachloroethylene (PCE)-contaminated drinking water: a retrospective cohort study
Source: Environ Health. 2011 Dec 2;10:102. doi: 10.1186/1476-069X-10-102 (PMC3268745; doi:10.1186/1476-069X-10-102)
Supplement: Additional file 8 — Table S8 Prenatal and Early Childhood Exposure to Tetrachloroethylene and Selected Risky Behaviors Among Subjects Without a History of Prenatal Exposure to Cigarette Smoke, Marijuana or Alcohol. [file 1476-069X-10-102-S8.DOC]

Table S8 Prenatal and Early Childhood Exposure to Tetrachloroethylene and Selected^1^ Risky Behaviors Among Subjects Without a History of Prenatal Exposure to Cigarette Smoke, Marijuana or Alcohol

Crude Simple GEE

Outcome Exposure % Yes (n/N) RR (95% CI) RR (95% CI)

Category/

Percentile

Ever smoked regularly vs. Never smoked regularly^2^ Any 29.6 (82/277) 1.0 (0.8-1.4) 1.0 (0.7-1.3)

>=67^th^ 38.1 (32/84) 1.3 (0.9-1.9) 1.3 (0.9-1.8)

33^rd^- <67^th^ 23.4 (25/107) 0.8 (0.5-1.2) 0.8 (0.5-1.2)

>0- <33^rd^  29.1 (25/86) 1.0 (0.7-1.5) 0.9 (0.6-1.4)

None 28.6 (55/192) Reference Reference

Smoked 20+ cigarettes a day vs. Never smoked regularly^3^ Any 9.7 (21/216) 1.4 (0.7-2.9) 1.4 (0.7-2.9)

>=67^th^ 11.9 (7/59) 1.7 (0.7-4.4) 1.7 (0.7-4.3)

33^rd^- <67^th^ 9.9 (9/91) 1.5 (0.6-3.4) 1.4 (0.6-3.4)

>0-<33^rd^ 7.6 (5/66) 1.1 (0.4-3.1) 1.2 (0.4-3.1)

None 6.8 (10/147) Reference Reference

First drank at <=13 years vs. 14+ years^4^ Any 16.9 (45/266) 1.2 (0.8-1.9) 1.2 (0.8-1.8)

>67^th^ 22.9 (19/83) 1.6 (0.9-2.7) 1.6 (0.9-2.8)

33^rd^- <67^th^  15.4 (16/104) 1.1 (0.6-1.9) 1.1 (0.6-1.9)

>0-<33^rd^ 12.7 (10/79) 0.9 (0.5-1.8) 0.9 (0.5-1.7)

None 14.2 (26/183) Reference Reference

Drank >8 days/mo as teen vs. Never drank as a teen^5^ Any 24.0 (23/96) 1.9 (1.0-3.7) 1.8 (0.9-3.7)

>67^th^ 36.7 (11/30) 2.9 (1.4-6.1) 2.9 (1.3-6.1)

33^rd^ - <67^th^  24.3 (9/37) 1.9 (0.9-4.3) 1.8 (0.8-4.2)

>0-<33^rd^ 10.3 (3/29) 0.8 (0.2-2.8) 0.8 (0.2-2.7)

None 12.7 (10/79) Reference Reference

Drank >=5/4 drinks/drinking day as teen Any 53.5 (84/157) 1.3 (1.0-1.7) 1.3 (1.0-1.7)

vs. Never drank as a teen^6^ >67^th^ 61.2 (30/49) 1.5 (1.1-2.1) 1.5 (1.1-2.0)

33^rd^ - <67^th^  51.7 (30/58) 1.3 (0.9-1.8) 1.2 (0.9-1.7)

>0-<33^rd^ 48.0 (24/50) 1.2 (0.8-1.7) 1.1 (0.8-1.6)

None 40.0 (46/115) Reference Reference

Table S8 Prenatal and Early Childhood Exposure to Tetrachloroethylene and Selected^1^ Risky Behaviors Among Subjects Without a History of Prenatal Exposure to Cigarette Smoke, Marijuana or Alcohol

Crude Simple GEE

Outcome Exposure % Yes (n/N) RR (95% CI) RR (95% CI)

Category/

Percentile

Drank >8 days in past 30 days Any 59.6 (84/141) 1.0 (0.8-1.3) 1.0 (0.8-1.2)

vs. Did not drink in past 30 days^7^ >67^th^ 74.4 (32/43) 1.3 (1.0-1.6) 1.2 (1.0-1.6)

33^rd^ - <67^th^  57.4 (27/47) 1.0 (0.7-1.3) 1.0 (0.7-1.3)

>0-<33^rd^ 49.0 (25/51) 0.9 (0.6-1.2) 0.8 (0.6-1.1)

None 57.4 (62/108) Reference Reference

Drank >=5/4 drinks/drinking day in past 30 days Any 38.7 (36/93) 1.1 (0.7-1.6) 1.1 (0.7-1.6)

vs. Did not drink in past 30 days^8^ >67^th^ 56.0 (14/25) 1.6 (1.0-2.5) 1.5 (1.0-2.4)

33^rd^ - <67^th^  35.5 (11/31) 1.0 ((0.6-1.7) 1.0 (0.6-1.7)

>0-<33^rd^ 29.7 (11/37) 0.8 (0.5-1.5) 0.8 (0.4-1.4)

None 36.1 (26/72) Reference Reference

Any drugs as a teen vs. Never used any drugs^9^ Any 59.0 (131/222) 1.1 (0.9-1.4) 1.1 (0.9-1.3)

>67^th^ 71.2 (47/166) 1.4 (1.1-1.7) 1.3 (1.1-1.6)

33^rd^ - <67^th^  53.5 (46/86) 1.0 (0.8-1.3) 1.0 (0.8-1.3)

0-<33^rd^ 54.3 (38/70) 1.0 (0.8-1.4) 1.0 (0.8-1.3)

None 52.2 (84/161) Reference Reference

2+ Drugs as a teen vs. Never used any drugs^9,10^ Any 39.7 (60/151) 1.3 (0.9-1.9) 1.3 (0.9-1.8)

>67^th^ 52.5 (21/40) 1.7 (1.2-2.6) 1.7 (1.1-2.6)

33^rd^ - <67^th^  36.5 (23/63) 1.2 (0.8-1.9) 1.2 (0.8-1.8)

>0-<33^rd^ 33.3 (16/48) 1.1 (0.7-1.8) 1.0 (0.6-1.7)

None 30.0 (33/110) Reference Reference

Any major drugs as a teen vs. Never used any drugs^9,11^ Any 40.1 (61/152) 1.3 (0.9-1.9) 1.3 (0.9-1.8)

>67^th^ 53.7 (22/41) 1.8 (1.2-2.7) 1.8 (1.2-2.7)

33^rd^ - <67^th^  36.5 (23/63) 1.2 (0.8-1.9) 1.2 (0.8-1.8)

0-<33^rd^ 33.3 (16/48) 1.1 (0.7-1.8) 1.0 (0.6-1.7)

None 30.0 (33/110) Reference Reference

Table S8 Prenatal and Early Childhood Exposure to Tetrachloroethylene and Selected^1^ Risky Behaviors Among Subjects Without a History of Prenatal Exposure to Cigarette Smoke, Marijuana or Alcohol

Crude Simple GEE

Outcome Exposure % Yes (n/N) RR (95% CI) RR (95% CI)

Category/

Percentile

2+ major drugs as a teen vs. Never used any drugs^9,12^ Any 26.0 (32/123) 1.4 (0.8-2.3) 1.4 (0.8-2.3)

>67^th^ 44.1 (15/34) 2.3 (1.3-4.1) 2.3 (1.3-4.1)

33^rd^ - <67^th^  21.6 (11/51) 1.1 (0.6-2.2) 1.1 (0.6-2.2)

>0-<33^rd^ 15.8 (6/38) 0.8 (0.4-1.9) 0.9 (0.4-2.0)

None 18.9 (18/95) Reference Reference

Marijuana as a teen vs. Never used any drugs^9,13^ Any 58.6 (129/220) 1.1 (0.9-1.4) 1.1 (0.9-1.3)

>67^th^ 70.3 (45/64) 1.3 (1.1-1.7) 1.3 (1.1-1.6)

33^rd^- <67^th^  53.5 (46/86) 1.0 (0.8-1.3) 1.0 (0.8-1.3)

>0-<33^rd^ 54.3 (38/70) 1.0 (0.8-1.4) 1.0 (0.8-1.3)

None 52.2 (84/161) Reference Reference

Inhalants as a teen vs. Never used any drugs^9,13^  Any 15.7 (17/108) 1.3 (0.6-2.5) 1.3 (0.6-2.5)

>67^th^ 26.9 (7/26) 2.2 (0.9-5.0) 2.1 (0.9-5.0)

33^rd^ - <67^th^  14.9 (7/47) 1.2 (0.5-2.9) 1.2 (0.5-2.9)

>0-<33^rd^ 8.6 (3/35) 0.7 (0.2-2.3) 0.7 (0.2-2.3)

None 12.5 (11/88) Reference Reference

Crack/cocaine as a teen vs. Never used any drugs^9,13^ Any 19.5 (22/113) 1.4 (0.8-2.8) 1.4 (0.7-2.8)

>67^th^ 38.7 (12/31) 2.9 (1.4-5.7) 2.8 (1.4-5.9)

33^rd^ - <67^th^  9.1 (4/44) 0.7 (0.2-2.0) 0.7 (0.2-2.0)

>0-<33^rd^ 15.8 (6/38) 1.2 (0.5-2.9) 1.2 (0.5-3.0)

None 13.5 (12/89) Reference Reference

Psychedelics/Hallucinogens as a teen vs. Never used any drugs^9,13^ Any 33.1 (45/136) 1.6 (1.0-.2.5) 1.6 (1.0-2.6)

67^th^ 45.7 (16/35) 2.2 (1.3-3.8) 2.3 (1.3-3.9)

33^rd^ - <67^th^  28.6 (16/56) 1.4 (0.8-2.4) 1.4 (0.8-2.6)

0-<33^rd^ 28.9 (13/45) 1.4 (0.8-2.6) 1.4 (0.7-2.6)

None 20.6 (20/97) Reference Reference

Table S8 Prenatal and Early Childhood Exposure to Tetrachloroethylene and Selected^1^ Risky Behaviors Among Subjects Without a History of Prenatal Exposure to Cigarette Smoke, Marijuana or Alcohol

Crude Simple GEE

Outcome Exposure % Yes (n/N) RR (95% CI) RR (95% CI)

Category/

Percentile

Club/Designer Drugs as a teen vs. Never used any drugs^9,13^ Any 18.8 (21/112) 1.4 (0.7-2.7) 1.4 (0.7-2.8)

>67^th^ 38.7 (12/31) 2.9 (1.4-5.7) 2.9 (1.4-6.0)

33^rd^ - <67^th^  14.9 (7/47) 1.1 (0.5-2.6) 1.1 (0.5-2.7)

>0-<33^rd^ 5.9 (2/34) 0.4 (0.1-1.8) 0.5 (0.1-1.9)

None 13.5 (12/89) Reference Reference

Ritalin as a teen vs. Never used any drugs^9,13^ Any 15.0 (16/107) 1.3 (0.6-2.7) 1.3 (0.6-2.7)

>67^th^ 32.1 (9/28) 2.8 (1.3-6.2) 2.8 (1.3-6.2)

33^rd^ - <67^th^  13.0 (6/46) 1.1 (0.4-2.9) 1.1 (0.4-2.9)

>0-<33^rd^ 3.0 (1/33) 0.3 (0.0-2.0) 0.3 (0.0-2.0)

None 11.5 (10/87) Reference Reference

Any Drugs as an adult vs. Never used any drugs^9^ Any 64.7 (167/258) 1.1 (1.0-1.3) 1.1 (0.9-1.3)

>67^th^ 76.3 (61/80) 1.3 (1.1-1.6) 1.3 (1.1-1.6)

33^rd^ - <67^th^  59.6 (59/99) 1.0 (0.9-1.3) 1.0 (0.8-1.3)

0-<33^rd^ 59.5 (47/79) 1.0 (0.8-1.3) 1.0 (0.8-1.3)

None 57.0 (102/179 ) Reference Reference

2+ Drugs as an adult vs. Never used any drugs^9,14^ Any 47.1 (81/172) 1.3 (1.0-1.8) 1.3 (1.0-1.8)

>67^th^ 61.2 (30/49) 1.7 (1.2-2.4) 1.7 (1.2-2.4)

33^rd^ - <67^th^  42.0 (29/69) 1.2 (0.8-1.7) 1.2 (0.8-1.7)

>0-<33^rd^ 40.7 (22/54) 1.2 (0.8-1.7) 1.1 (0.7-1.7)

None 35.3 (42/119) Reference Reference

Major Drugs as an adult vs. Never used any drugs^9,15^  Any 48.6 (86/177) 1.3 (1.0-1.7) 1.2 (0.9-1.6)

>67^th^ 62.7 (32/51) 1.6 (1.2-2.2) 1.6 (1.2-2.2)

33^rd^ - <67^th^  42.9 (30/70) 1.1 (0.8-1.6) 1.1 (0.8-1.6)

0-<33^rd^ 42.9 (24/56) 1.1 (0.8-1.6) 1.1 (0.7-1.6)

None 38.4 (48/125) Reference Reference

Table S8 Prenatal and Early Childhood Exposure to Tetrachloroethylene and Selected^1^ Risky Behaviors Among Subjects Without a History of Prenatal Exposure to Cigarette Smoke, Marijuana or Alcohol

Crude Simple GEE

Outcome Exposure % Yes (n/N) RR (95% CI) RR (95% CI)

Category/

Percentile

2+ Major Drugs as an adult vs. Never used any drugs^9,16^ Any 36.8 (53/144) 1.7 (1.1-2.5) 1.6 (1.0-2.5)

>67^th^ 48.6 (18/37) 2.2 (1.3-3.6) 2.1 (1.3-3.5)

33^rd^ - <67^th^  35.5 (22/62) 1.6(1.0-2.6) 1.5 (0.9-2.5)

>0-<33^rd^ 28.9 (13/45) 1.3 (0.7-2.3) 1.3 (0.7-2.3)

None 22.2 (22/99) Reference Reference

Marijuana as an adult vs. Never used any drugs^9,17^ Any 63.7 (160/251) 1.1 (1.0-1.4) 1.1 (0.9-1.3)

>67^th^ 75.6 (59/78) 1.4 (1.1-1.6) 1.3 (1.1-1.6)

33^rd^- <67^th^  58.3 (56/96) 1.1 (0.8-1.3) 1.0 (0.8-1.3)

>0-<33^rd^ 58.4 (45/77) 1.1 (0.8-1.3) 1.0 (0.8-1.3)

None 55.5 (96/173) Reference Reference

Crack/cocaine as an adult vs. Never used any drugs^9,17^  Any 35.9 (51/142) 1.4 (1.0-2.1) 1.4 (0.9-2.1)

>67^th^ 51.3 (20/39) 2.0 (1.3-3.2) 2.0 (1.2-3.1)

33^rd^ - <67^th^  28.6 (16/56) 1.1 (0.7-1.9) 1.1 (0.6-1.9)

>0-<33^rd^ 31.9 (15/47) 1.3 (0.7-2.2) 1.3 (0.7-2.2)

None 25.2 (26/103) Reference Reference

Psychedelics/Hallucinogens as an adult Any 31.1 (41/132) 1.2 (0.8-1.9) 1.2 (0.8-1.9)

vs. Never used any drugs^9,17^ >67^th^ 36.7 (11/30) 1.5 (0.8-2.6) 1.5 (0.8-2.7)

33^rd^ - <67^th^  31.0 (18/58) 1.2 (0.7-2.0) 1.2 (0.7-2.0)

>0-<33^rd^ 27.3 (12/44) 1.1 (0.6-1.9) 1.1 (0.6-2.0) None 25.2 (26/103) Reference Reference

Club/Designer Drugs as an adult vs. Never used any drugs^9,17^ Any 38.5 (57/148) 1.9 (1.2-2.9) 1.8 (1.2-2.8)

>67^th^ 48.6 (18/37) 2.4 (1.4-3.9) 2.3 (1.4-3.9)

33^rd^ - <67^th^  39.4 (26/66) 1.9 (1.2-3.1) 1.8 (1.1-3.0)

>0-<33^rd^ 28.9 (13/45) 1.4 (0.8-2.6) 1.4 (0.7-2.5)

None 20.6 (20/97) Reference Reference

Table S8 Prenatal and Early Childhood Exposure to Tetrachloroethylene and Selected^1^ Risky Behaviors Among Subjects Without a History of Prenatal Exposure to Cigarette Smoke, Marijuana or Alcohol

Crude Simple GEE

Outcome Exposure % Yes (n/N) RR (95% CI) RR (95% CI)

Category/

Percentile

Ritalin as an adult vs. Never used any drugs^9,17^ Any 22.9 (27/118) 1.4 (0.8-2.5) 1.4 (0.8-2.5)

>67^th^ 38.7 (12/31) 2.4 (1.3-4.5) 2.4 (1.2-4.5)

33^rd^ - <67^th^  18.4 (9/49) 1.1 (0.5-2.4) 1.1 (0.5-2.3)

>0-<33^rd^ 15.8 (6/38) 1.0 (0.4-2.3) 1.0 (0.4-2.3)

None 16.3 (15/92) Reference Reference

1 Table excludes outcomes with five or fewer subjects in the referent category

2 Comparison excludes subjects who smoked 100+ cigarettes but never became regular smokers

3 Comparison excludes subjects who smoked < 20 cigarettes a day

4 Comparison excludes subjects who never drank as a teen

5 Comparison excludes subjects who drank <= 8 days/month as a teen

6 Comparison excludes subjects who drank <5/4 drinks/drinking day as a teen

7 Comparison excludes subjects who drank <= 8 days/month in past 30 days

8 Comparison excludes subjects who drank <5/4 drinks/drinking day in past 30 days

9 Referent group is comprised of subjects who never used drugs as a teen or an adult

10 Comparison excludes subjects who used only one drug as a teen

11 Comparison excludes subjects who used only marijuana as a teen

12 Comparison excludes subjects who used only marijuana or one major drug as a teen

13 Comparison excludes subjects who used any other type of drug as a teen

14 Comparison excludes subjects who used only one drug as an adult

15 Comparison excludes subjects who used only marijuana as an adult

16 Comparison excludes subjects who used only marijuana or one major drug as an adult

17 Comparison excludes subjects who used any other type of drug as an adult
